# Effect of Dietary Microalgae (*Spirulina platensis*) on Growth Performance, Ingestive Behavior, Hemato-Biochemical Parameters, and Economic Efficiency of Fayoumi Broilers

**DOI:** 10.3390/life12111892

**Published:** 2022-11-15

**Authors:** Rasha I. M. Hassan, Mohamed S. Refaie, Ramadan D. El-Shoukary, Ibrahim F. Rehan, František Zigo, Viera Karaffová, Hala Y. Amer

**Affiliations:** 1 Department of Nutrition and Clinical Nutrition, Faculty of Veterinary Medicine, Assiut University, Assiut 71515, Egypt; 2 Department of Poultry Production, Faculty of Agriculture, New Valley University, El-Kharga 72511, Egypt; 3 Department of Animal Wealth Development, Faculty of Veterinary Medicine, New Valley University, El-Kharga 72511, Egypt; 4 Department of Husbandry and Development of Animal Health, Faculty of Veterinary Medicine, Menoufia University, Shibin el Kom 32511, Egypt; 5 Department of Pathobiochemistry, Faculty of Pharmacy, Meijo University, Yagotoyama 150, Tempaku-ku, Nagoya-shi 468-8503, Japan; 6 Department of Nutrition and Animal Husbandry, University of Veterinary Medicine and Pharmacy, Komenského 73, 04181 Košice, Slovakia; 7 Department of Morphological Disciplines, University of Veterinary Medicine and Pharmacy in Košice, 04181 Košice, Slovakia; 8 Department of Animal Nutrition and Clinical Nutrition, Faculty of Veterinary Medicine, New Valley University, El-Kharga 72511, Egypt

**Keywords:** *Spirulina platensis*, growth performance, blood parameters, carcass characteristics, Fayoumi broiler

## Abstract

This study was conducted to evaluate the effect of dietary supplementation with *Spirulina platensis* (SP) on the productive performance, carcass characteristics, behavior, blood serum metabolites, hematological indices, and economic efficiency of Fayoumi broiler chickens for a 56-day. In total, 120 one-day-old broiler chicks were randomly distributed among four dietary treatments with three replicates (n = 10/group) for 8 weeks. The dietary treatments were a control basal diet without SP and the same basal diets supplemented with 0.25, 0.5, or 1.0% SP. Birds fed 1% *Spirulina*-supplemented diets recorded significantly (*p* < 0.05) higher body weight, weight gain, and feed conversion ratio and less overall feed intake and feeding behavior than those in the control group. No significant changes (*p* > 0.05) were recorded in the dressing percentage or the relative weights of internal organs among the different experimental groups, except for the thymus. Diets containing 0.5 or 1.0% SP saw an increase (*p* < 0.05) in serum total protein and globulin and a reduction (*p* < 0.05) in serum cholesterol concentration. The lymphocyte percentage in birds fed SP diets was significantly (*p* < 0.05) higher than in birds fed the control diet. These results suggest that adding SP up to 1% to the broiler diets could positively affect some important blood biochemical parameters, enhance their immunity response, and improve their growth performance. However, from an economic point of view, supplementation with 0.25% of SP is recommended for Fayoumi broiler chickens.

## 1. Introduction

For many years, antibiotic growth promoters (AGPs) have been widely used in poultry diets. Improvement of the bird’s productive performance is the main effect of AGP, in addition to preventing, controlling, and reducing diseases and mortalities caused by poultry pathogens [1]. Unfortunately, the misuse of AGP and the development of drug-resistant bacteria diminished its use in poultry diets [2]. To overcome this problem, finding alternatives to AGPs was the best solution. Different kinds of phytogenic feed additives, especially marine plants, herbs, and other natural materials, can be used as AGP alternatives in poultry diets without any negative effect on bird or human health. Because of their antioxidant, anti-inflammatory, antibacterial, and immune-modulatory activities, these phytogenic compounds can improve birds’ growth performance and enhance their immune response [3]. One of these natural compounds is *Spirulina platensis.* In *Spirulina platensis* (SP, cyanobacteria), the major bioactive component is phycocyanin (PC), which is a blue photosynthetic pigment in cyanobacteria and some red algae of the phycobiliprotein family. It is water-soluble, located in the cytoplasmic membrane, and released outside when the thylakoid membrane is destroyed by the lysozyme enzyme and EDTA chelate cations. *Spirulina platensis* has been used as a human and animal food source for hundreds of years [4].

It is considered a superfood due to its richness in protein, vitamins (B1, B2, B12, and vitamin C), minerals (calcium, phosphorus, iron, selenium, and copper), amino acids, essential fatty acids, and xanthophyll phytopigments [5]. Dried SP contains about 50–65% protein, 12–13% carbohydrate, and 6% fat [6,7]. It is used in poultry diets to modulate the defense system mechanisms by increasing the ability to kill pathogenic microbes and increasing the T cells’ activities [8]. *Spirulina* has been reported to have antimicrobial, anti-inflammatory, anticancer, antiviral, antioxidant, immune-stimulating, antibacterial, hypocholesterolemia, and metalloprotective effects [9,10]. The powerful antioxidant effect of SP is related to its content of bioactive compounds such as flavonoids, carotenoids, γ-linoleic acid, phenolic compounds, phycocyanin, vitamin C, and selenium, which act as natural antioxidant compounds [11]. The algae supplementation of SP can improve body weight gain, feed conversion ratio, serum lipid profile, lymphoid organ weights, and leucocyte count, and decrease mortality rates in broiler chickens [12,13]. Although the inclusion of SP in poultry diets shows a promising future, the optimum range of SP inclusion has not yet been identified in the Fayoumi broiler diet. Therefore, this experiment aims to evaluate the effects of different levels of SP inclusion in feed on the growth performance, carcass characteristics, ingestive behavior, serum biochemical parameters, blood profile, and cost-effectiveness in Fayoumi broiler birds.

## 2. Materials and Methods

### 2.1. Ethical Approval

The current work was carried out according to the regulations and procedures approved by the ethics committee on animal experimentation at New Valley University, Faculty of Veterinary Medicine, and the guide for the care and use of animals (National Institute of Health Publication No. 8023, revised 1978).

### 2.2. Birds, Experimental Design and Husbandry

**Figure 1:**
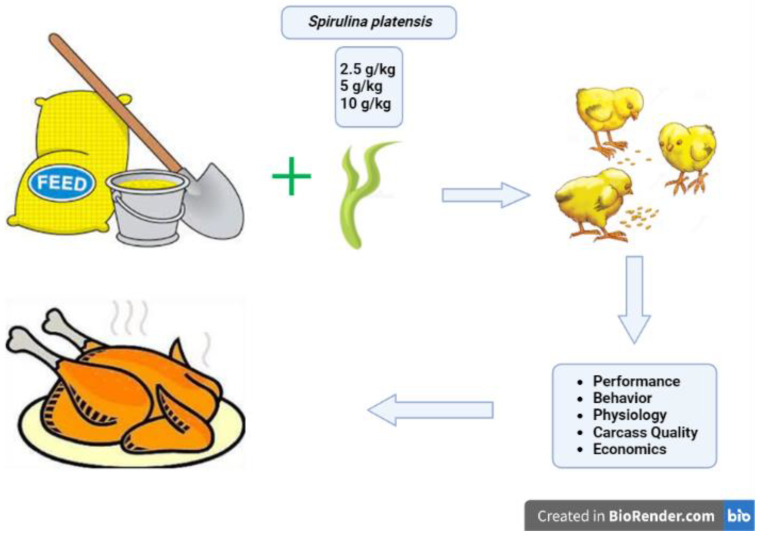
The schematic cartoon of the experimental design—it was created with BioRender.com (accessed on 2 October 2022).

A total of 120 one-day-old Fayoumi chicks were obtained from a local commercial source (Poultry Farm, Assiut, Egypt), weighed, and randomly divided into four groups with three replicates of 10 birds each, based on a completely randomized design. The birds were reared in separate floor pens with a wood shaving floor and had free access to feed and water. During the 56 days of the experimental period, environmental factors such as temperature, lighting, humidity, and ventilation were maintained at optimal levels according to the commercial recommendations. Treatment 1 (control) was fed a basal diet only, and treatments 2–4 were fed a basal diet supplemented with 2.5, 5, and 10 g/kg *Spirulina platensis*, respectively. All experimental chicks were fed mash diets formulated according to the requirements proposed in [14,15,16]. A starter diet with 22% crude protein (CP) and a 3071 kcal ME/ kg diet from days 1 to 28, and a grower–finisher diet with 19% CP and a 3150 kcal ME/kg diet from days 29 to 56 were offered (Table 1). The experimental strategy is shown in a schematic cartoon (Figure 1).

### 2.3. Spirulina platensis

The *Spirulina platensis* (SP) used in this experiment was purchased from a commercial company (Imtenan, Assiut, Egypt). The chemical analysis of SP was conducted in the laboratory of the Department of Animal Nutrition and Clinical Nutrition, Faculty of Veterinary Medicine, New Valley University, Egypt, according to the methods described by the Association of Official Analytical Chemists [17]. The chemical composition was 88.50% dry matter (DM, AOAC official method 930.15), 56.40% crude protein (CP, AOAC official method 984.13), 7.5% ether extract (EE, AOAC official method 920.39), 3.50% crude fiber (CF, AOAC method 978.10), and 9.30% ash (AOAC official method 942.05).

### 2.4. Bird Performance

Body weights of the Fayoumi chicks were measured at 1, 14, 28, 42, and 56 days, and average feed intake was determined for the different experimental periods. The feed conversion ratio was calculated subsequently based on the body weight gain and feed consumption during the same period.

### 2.5. Hemato-Biochemical Analysis

For hematology, heparinized tubes were used to collect the blood samples. The whole blood picture included red blood cells, hemoglobin, packed cell volume, mean corpuscular volume, mean corpuscular hemoglobin, mean corpuscular hemoglobin concentration, and white blood cell count. Lymphocytes and monocytes were estimated using a hemocytometer slide. For biochemical analysis, serum was collected in tubes free of anticoagulant, centrifuged at 805× *g* force for 15 min, and kept at −20 °C until used to estimate total proteins, albumin, triglycerides, and cholesterol using commercial kits (Egyptian Company for Biotechnology, Cairo, Egypt).

### 2.6. Behavioral Observations

Fayoumi broilers were directly observed in two periods of the day, in the morning (7:00–9:00 a.m.) and in the afternoon (1:00–3:00 p.m.), for two inconsecutive days/week for nine weeks, corresponding with a technique reported previously [16]. Each period was 15 min long and was analyzed via the scanning technique [18]. The number of chicks engaged in ingestive behavior was observed and recorded (act/30 min) (i.e., feeding: bird head observed inside the feeder and drinking bird head in contact with water).

### 2.7. Carcass Evaluation

On day 56, three birds from each group were randomly selected, weighed, and slaughtered for carcass evaluation. Proventriculus, gizzard, liver, heart, spleen, thymus, and bursa were excised and weighed after slaughtering. The following formula calculates the dressing percentage: Dressing % = (carcass weight/live weight) × 100.

### 2.8. Economical Assessment

This trial’s economic evaluation was calculated using the feed and production costs, the gross return, and the net return. The feed cost includes the cost of rations and additives, while the production cost includes the cost of birds purchased, feeding costs, veterinary care, and other husbandry costs. The gross return is the selling price of the birds at the end of the experiment. The net return is the difference between the gross return and the cost. In addition, the economic efficiency (net return divided by total cost) and relative economic efficiency (economic efficiency of group/economic efficiency of control*100) were calculated according to the previous report [19].

### 2.9. Statistical Analyses

Collected data were statistically analyzed using the Statistical Package for Social Science (SPSS 16.0). The Duncan’s Multiple Range Test was used to compare treatment means, and the significance level was considered at the 5% level [20]. The statistical model used for the trial was Y_ij_ = μ + T_i_ + E_ij_, where Y_ij_ = response variables; *μ* = the overall mean; T_i_ = the effect of dietary treatment; and E_ij_ = the experimental error.

## 3. Results

### 3.1. Growth Performance Finding

The effects of various dietary inclusions of *Spirulina platensis* (SP) on the growth performance indices of the birds are presented in Table 2. The results showed no significant (*p* > 0.05) difference in broilers’ body weight and weight gain between experimental groups up to 6 weeks of age. However, the birds fed diets supplemented with 0.25 or 0.5% SP had numerically greater body weight than those provided with the control diet. In addition, the body weight of birds supplemented with SP at 1% was significantly (*p* < 0.05) higher than in the control group on week 8. However, there was no significant difference in live body weight at 8 weeks between the SP-supplemented groups. Birds fed diets with 0.25 or 0.5% SP had numerically higher total weight gain than those fed the control diet. Birds fed diets supplemented with SP at 1% had a significantly (*p* < 0.05) higher total weight gain than those fed the control diet. However, no significant differences were detected in total gain between the SP-supplemented groups. The cumulative feed intake from day 1 to 56 was numerically decreased for birds supplemented with SP. The lowest significant (*p* < 0.05) feed intake was recorded in birds fed 1% SP-supplemented diets. However, the feed intake of the birds did not differ significantly (*p* > 0.05) between the treated and control group until 4 weeks of age. The feed conversion ratio (FCR) did not show any significant (*p* > 0.05) difference between groups in weeks 4–6. However, at 8 weeks, there was a significant (*p* < 0.05) decrease in the FCR of birds in SP groups compared to the control. In addition, the groups fed 0.5 and 1% SP recorded significantly lower (*p* < 0.05) values of total FCR compared to other treatments. The best total FCR was recorded in birds fed 1% SP diets.

### 3.2. Hemato-Biochemical Indices

Serum total protein and globulin were increased (*p* < 0.05) in birds fed 0.5 or 1% SP, as compared to those fed 0.25% SP or the control diet (Table 3). However, no significant (*p* > 0.05) differences were recorded in serum albumin and triglycerides among the treated groups. The serum cholesterol concentration decreased (*p* < 0.05) as the level of SP increased. Moreover, the results indicated no significant differences due to the supplementation of SP in the Fayoumi broilers’ diet on all hematological parameters, except for WBCs and lymphocytes (Table 3). The birds fed diets containing SP (0.5%) had higher WBCs (*p* < 0.05) than birds in other treated groups. All dietary supplements from SP significantly (*p* < 0.05) increased the lymphocyte percentage.

### 3.3. Behavioral Findings

**Figure 2:**
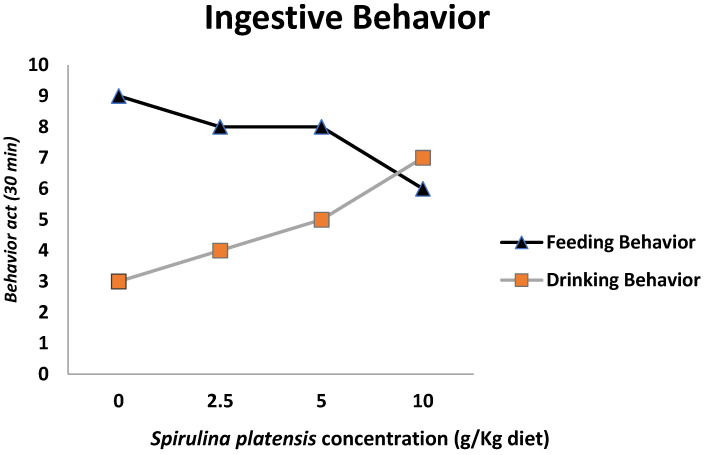
Effects of dietary treatments *Spirulina platensis* concentration (g/Kg diet) on the ingestive behavior of Fayoumi broiler chickens.

There were increases in the drinking behavior and decreases in the feeding behavior of Fayoumi broilers due to the *Spirulina platensis* addition, as shown in Figure 2.

### 3.4. Carcass Characteristics

The effects of dietary supplementation with SP on carcass characteristics and the relative weight of immune organs are shown in Table 4. No significant differences were detected in dressing percentage or the relative weights of the proventriculus, gizzard, heart, liver, bursa, and spleen among the four treatment groups (*p* > 0.05). However, thymus % was significantly (*p* < 0.05) higher in birds fed 0.5% SP compared to the 0.25% SP-supplemented diets and control group.

### 3.5. Economic Impact

The results of the economic evaluation of the different experimental diets over a 56-day period as affected by *Spirulina platensis* levels are presented in Table 5. It is obvious that including SP in the Fayoumi broiler diets increased the feed cost as compared to that of the control diet. The production cost was lower for the control treatment and was highest for the T4 treatment due to the cost of SP added to the ration. The gross return was higher in T4, followed by T3 and T2, and the lowest gross return was recorded for the SP-free diets. Similarly, the net return was higher in all SP groups as compared with the control group. The best economic efficiency and relative economic efficiency values were recorded in T2, whereas the poorest values were recorded in T4.

## 4. Discussion

### 4.1. Influence of Dietary Spirulina platensis on Performance and Ingestive Behavior of Broilers

Incorporating microalgae into the birds’ diet offers a great chance to improve their growth performance, but the results extensively depend on the types, chemical composition, and inclusion levels of algae in animal diets [21]. The significant improvement in the birds’ growth performance parameters (body weight, weight gain, and feed conversion) fed SP-containing diets may be attributed to the fact that the protein content of SP is rich in essential amino acids (EAAs). These EAAs, such as methionine, lysine, tryptophan, glutamic acid, and aspartic acid, have a significant role in the development of intestinal mucous membranes, leading to an increase in villus height and crypt depth, which reflect greater nutrient absorption [22]. The physiologically active compounds in SP include polyunsaturated fatty acids, beta-carotene, phenols, phycocyanin, water- and fat-soluble vitamins, and minerals such as phosphorus and copper. These compounds have potent antimicrobial, antioxidant, and immune-enhancing properties [23]. Moreover, they have the ability to enhance beneficial intestinal microbiota growth as a result of lactobacilli, which plays a significant role in the growth and immune responses of birds through the enhancement of dietary vitamin and mineral absorption and availability [24]. The same results were recorded by other researchers, who showed that the birds fed 1% SP diets achieved superior live body weights, weight gains, and feed conversion ratios compared to those of the control groups [25,26]. Moreover, a significant increase (*p* < 0.001) in live body weight was recorded between broilers fed SP-supplemented diets at levels of 5 and 10 g/kg and those in the control group [27]. Furthermore, diet supplementation with 1% SP significantly (*p* < 0.05) improved the live weight, weight gain, and feed conversion ratio of broilers compared with birds that were fed non-supplemented diets on days 28 and 35 [28]. On the other hand, the decrease in the feeding behavior of Fayoumi with the increase in SP addition disagrees with the finding of the previous report [29], which recorded that there were no significant differences in total feed intake and accumulative feed conversion rate between broiler birds fed 1% SP-supplemented diets and control birds. Finally, the increase in drinking behavior may be due to the high protein, vitamin and mineral, carotene, and xanthophyll phytopigment content of SP [5].

### 4.2. Influence of Dietary Spirulina platensis on Carcass Traits

Regarding the positive effect of SP at 0.5% on thymus relative weight, our results are consistent with the findings of other researchers [30,31]. They reported that the dietary supplementation of *Spirulina* in chicken diets at less than 1% significantly stimulates T-cell-mediated immune responses and enhances microbial killing activities. In addition, the high content of Zn in *Spirulina* significantly promotes the cellular immunity of broiler birds. On the other hand, supplementing broiler diets with different levels of SP did not affect the relative weight of the gizzard, heart, liver, bursa, or spleen [22,26]. Our results are inconsistent with other findings obtained previously [32]. They noticed that the relative weights of the bursa and spleen were significantly (*p* < 0.05) improved with SP supplementation.

### 4.3. Influence of Dietary Spirulina platensis on the Biochemical Parameters of Fayoumi Broilers

As the serum protein depends on the availability of dietary crude protein, the proteins of SP-supplemented diets were more available to the broiler birds. The increase in serum globulin cleared in the present trial from feeding broiler rations containing SP at 0.5 and 1% might be related to the immune-stimulating effect of SP. The hypo-cholesterolaemic effect of SP could be attributed to both the reduction in the absorption and synthesis of cholesterol in the intestinal tract and the enhancement in the growth and multiplication of the lactobacilli population [33]. In addition, the bioactive components in *Spirulina*, such as phenolic compounds, linolenic acid, and *C*-phycocyanin protein, have cholesterol-lowering properties [34]. The present results agree with those obtained in other studies and demonstrate significantly increased serum globulin and decreased total serum cholesterol in broilers fed 0.5% SP-supplemented diets [32,35]. In addition, it was noticed that serum albumin and triglycerides were not affected by supplementing SP in broiler diets [36].

### 4.4. Influence of Dietary Spirulina platensis on the Physiological Parameters of Fayoumi Broilers

The richness of SP, with trace minerals such as iron, copper, and zinc, may explain the improvements in blood hematology. In addition, the maturation and differentiation of T-lymphocytes, which are responsible for the birds’ cellular immune responses, may be related to the same cause. Moreover, the bioactive components of *Spirulina*, such as phycocyanin and polysaccharides, have a significant role in the development and maturation of WBCs, which help to improve humoral and cellular immune responses in birds [37,38]. A significant increase in the white blood cells of broiler chickens fed *Spirulina*-supplemented diets was observed [39]. Furthermore, a significant (*p* < 0.05) increase in RBC count and hemoglobin values was observed in the blood of broilers fed *Spirulina*-supplemented diets at 2–8 g/kg feed [40].

### 4.5. Influence of Dietary Spirulina platensis on the Economics of Fayoumi Broiler Farm

The current study showed that feeding SP to broiler birds improved the gross and net returns of broiler farming. However, both economic efficiency and relative economic efficiency values were lower due to SP at higher levels (0.5 and 1%) and the higher cost of SP. In our study, the most cost-effective level of SP in broiler diets was 0.25%. Our findings agree with a previous finding [32], in which the authors recorded that the inclusion levels of SP in diets at 0.3–0.9 of feed were cost-effective with regard to significant improvements in broiler health and growth performance. It was reported that SP supplementation in broiler diets has a positive effect on gross and net return values [41]. Despite the significant improvement in broiler carcass performance due to the dietary addition of *Spirulina platensis* at a level above 1% [42], the high cost of the SP product diminishes the profitability of broiler farming.

## 5. Conclusions

The dietary supplementation of SP at 1% to Fayoumi broiler chicken diets positively affects the birds’ performance and immunity and negatively affects feeding behavior frequency and serum cholesterol levels. However, from an economic point of view, the most cost-effective level of SP in the Fayoumi broiler diet is 0.25%.

## Figures and Tables

**Table 1:** Ingredients (%) and nutrient composition of basal diets of Fayoumi broilers.

Item	Starter	Grower–Finisher
Yellow corn	58.8	66.2
Soybean meal, 48% CP	32.1	26.4
Sunflower oil	3.8	4.18
Corn gluten	1.32	0.00
Di-calcium phosphate	1.78	1.02
Limestone, ground	1.35	1.56
Methionine	0.15	0.02
Lysine	0.1	0.02
Salt	0.3	0.3
Premix *	0.3	0.3
**Total (%) Calculated Analysis**	100	100
Crude protein	22	19
Ether extract	6.58	7.07
Crude fiber	3.36	3.13
Calcium	1.0	0.9
Available phosphorus	0.45	0.3
Lysine	1.13	0.9
Methionine	0.5	0.32
**Energy content:**		
ME (kcal/kg diet)	3071	3150

* Each kg contains: Vit. A, 6250000 IU; Vit. D3, 2500000 IU; Vit. E, 25,000 mg; Vit. k3, 1750 mg; Vit. B1, 500 mg; Vit. B2, 2750 mg; Vit. B6, 1250 mg; Vit. B12, 10 mg; nicotinic acid 20,000 mg; calcium pantothenate, 500 mg; folic acid 500 mg; biotin 50 mg; iron 22g; copper 2.5 g; zinc 37.5 g; manganese 31 g; iodine 650 mg; selenium 113 mg; cobalt 50 mg.

**Table 2:** Effects of dietary treatments (*Spirulina platensis*) on the growth performance of Fayoumi broilers.

Item	Dietary Treatments					
	T1	T2	T3	T4	SEM	*p*-Value
**Body weight (g/bird)**						
Initial	35.42	35.17	35.17	35.25	0.565	0.988
2 weeks	141.58	149.75	138.5	140	4.21	0.248
4 weeks	342.08	356.67	360	351.67	11.59	0.716
6 weeks	585	614.17	629.25	608.42	25.31	0.666
8 weeks	866.67 ^b^	925.83 ^ab^	932.08 ^ab^	976.08 ^a^	33.58	0.163
**Body weight gain (g/bird)**						
0–2 weeks	106.17	114.58	103.33	104.75	3.849	0.176
2–4 weeks	200.5	206.92	221.5	2.1167	8.238	0.339
4–6 weeks	242.92	257.5	269.25	258	15.247	0.683
6–8 weeks	281.67 ^b^	311.67 ^b^	302.83 ^b^	366.42 ^a^	12.606	0
**Overall**	831.25 ^b^	890.67 ^ab^	896.92 ^ab^	940.83 ^a^	33.153	0.153
**Feed intake (g/bird)**						
0–2 weeks	106	106.67	113.33	102	3.613	0.246
2–4 weeks	297	292.66	308.67	291	8.36	0.479
4–6 weeks	636.66 ^a^	635.00 ^a^	599.00 ^b^	590.00 ^b^	7.63	0.005
6–8weeks	800.00 ^a^	797.66 ^a^	809.83 ^a^	675.67 ^b^	6.857	0
**Overall**	1840 ^a^	1832 ^a^	1831 ^a^	1659 ^b^	26.01	0.003
**Feed conversion% (g/g)**						
0–2 weeks	1.01 ^ab^	0.93 ^b^	1.08 ^a^	0.98 ^ab^	0.035	0.034
2–4 weeks	1.48	1.42	1.37	1.39	0.05	0.419
4–6 weeks	2.67	2.56	2.24	2.45	0.147	0.215
6–8 weeks	2.88 ^a^	2.61 ^b^	2.63 ^ab^	1.86 ^c^	0.092	0
**Overall**	2.23 ^a^	2.08 ^ab^	2.02 ^b^	1.79 ^c^	0.072	0.001

Means within the same row, with different superscripts, are significantly different (*p* < 0.05). T1, T2, T3, and T4 are the control group (0.0), 0.25, 0.5, and 1% *Spirulina*, respectively.

**Table 3:** Effects of dietary treatments (*Spirulina platensis*) on some serum metabolites and blood hematological parameters of Fayoumi broilers.

Item	Dietary Treatments					
	T1	T2	T3	T4	SEM	*p*-Value
**Serum metabolites**						
Total protein, g/dL	3.35 ^b^	3.24 ^b^	4.98 ^a^	5.44 ^a^	0.261	0.001
Albumin, g/dL	2.32	2.38	2.09	2.07	0.119	0.17
Globulin, g/dL	0.95 ^b^	1.04b	2.88 ^a^	3.37 ^a^	0.316	0.001
Triglycerides, mg/dL	61.54	60.23	67.98	63.94	6.22	0.825
Cholesterol, mg/dL	1.07 ^a^	1.01 ^ab^	93.52 ^b^	90.85 ^b^	3.99	0.07
**Hematological parameters**						
RBCs (×10^6^/mm^3^)	4.47	4.33	4.26	4.56	0.13	0.416
Hemoglobin, g/dL	7.9	7.8	6.51	6.93	0.573	0.318
PCV, %	22.7	22.66	19.46	20.3	1.38	0.306
MCV, fl	52.66	52.33	49.66	50	1.47	0.4
MCH, pg	17.23	17.33	16.33	16.33	0.869	0.757
MCHC, %	34.53	34.4	34.23	34.43	0.194	0.747
WBCs (×10^3^/mm^3^)	9.46 ^b^	9.40 ^b^	11.75 ^a^	7.80 ^b^	0.599	0.011
Lymphocyte %	77.00 ^b^	80.00 ^a^	81.00 ^a^	80.00 ^a^	0.898	0.043
Monocyte %	2.33	1.33	2.33	1.67	0.3	0.111

Means within the same row with different superscripts are significantly different (*p* < 0.05). T1, T2, T3 and T4 are control group (0.0), 0.25, 0.5, and 1% *Spirulina,* respectively. SEM, pooled standard error of the mean RBC, red blood cell; Hb, hemoglobin; PCV, packed cell volume; MCV, mean corpuscular volume; MCH, mean corpuscular hemoglobin; MCHC, mean corpuscular hemoglobin concentration; WBC, white blood cell.

**Table 4:** Effects of dietary treatments (*Spirulina platensis*) on carcass traits and some immune organs of Fayoumi broilers.

Item	Dietary Treatments					
	T1	T2	T3	T4	SEM	*p*-Value
**Carcass traits (%)**						
Dressing	68.95	69.09	69.45	69.34	0.154	0.164
Proventriculus	0.85	0.86	0.86	0.81	0.068	0.928
Gizzard	2.29	2.29	2.4	2.42	0.059	0.309
Heart	0.54	0.54	0.56	0.57	0.019	0.66
Liver	2.14	2.18	2.19	2.22	0.029	0.317
**Immune organs (%)**						
Thymus	0.51 ^b^	0.51 ^b^	0.64 ^a^	0.59 ^ab^	0.025	0.013
Bursa	0.03	0.03	0.04	0.04	0.005	0.119
Spleen	0.22	0.22	0.3	0.29	0.018	0.017

Means within the same row, with different superscripts, are significantly different (*p* < 0.05). T1, T2, T3, and T4 are the control group (0.0), 0.25, 0.5 & 1% *Spirulina*, respectively.

**Table 5:** Effect of dietary levels of *Spirulina platensis* on Fayoumi broilers’ economic efficiency.

Items	Dietary Treatments			
	T1	T2	T3	T4
Feed cost/bird (kg)	9.5	10.25	11	12.5
Production cost (LE)	20.48	21.78	23.14	23.74
Gross return (LE)	41.62	44.45	44.74	46.85
Net return (LE)	21.14	22.67	21.6	23.11
Economic efficiency	103.2	104.09	93.31	97.35
Relative economic efficiency	100	100.86	90.42	94.33

T1, T2, T3 and T4 are the control group (0.0), 0.25, 0.5 and 1% *Spirulina*, respectively. LE: Egyptian pound. Economic efficiency: Net return per unit production cost. Relative economic efficiency: Economic efficiency of group/Economic efficiency of control*100.

## Data Availability

The data that support their conclusions are available from the authors of this manuscript upon request.
